# The suppressive effect of co-inhibiting *PD-1* and *CTLA-4* expression on H22 hepatomas in mice

**DOI:** 10.1186/s11658-018-0122-0

**Published:** 2018-12-15

**Authors:** Leilei Liang, Keli Ge, Fengying Zhang, Yinlin Ge

**Affiliations:** 1 Department of Biochemistry and Molecular Biology, Medical College, Qingdao University, 38 Dengzhou Road, Qingdao, 266021 Shandong China; 2 Central Laboratory, Binzhou People’s Hospital, Binzhou, 256610 Shandong China; 3 Integrative Medicine Research Center, Medical College, Qingdao University, Qingdao, 266021 Shandong China; 4 Department of Biochemistry and Molecular Biology, Heze Medical College, Heze, 274000 Shandong China

**Keywords:** PD-1, CTLA -4, H22, siRNA, Immune, IFN-γ

## Abstract

**Objective:**

We investigated the suppressive effect of siRNA-mediated co-inhibition of *PD-1* and *CTLA-4* expression on H22 hepatomas in mice.

**Methods:**

Murine H22 cells were cultured in vivo in ICR mice. An allograft tumor model was also established in another ICR mouse group. The tumor-bearing mice were randomly divided into four groups: control, single *PD-1* siRNA, single *CTLA-4* siRNA, and double *PD-1 + CTLA-4* siRNAs. The survival time and physiological condition of the mice were observed after the injection of the siRNAs and placebo. The volume and weight of the solid tumor were measured to assess the inhibition of the tumor. To assess the effects of siRNAs on mouse immune function, the protein levels of IFN-γ and IL-10 in the blood and PD-L1 in the tumor and liver were determined using ELISA, and the mRNA levels of *IFN-γ*, *PD-L1*, *PD-1*, *CTLA-4*, *IL-6* and *Survivin* in the tumor, liver and spleen were determined using quantitative RT-PCR. The ratios of Bax and Bcl-2 protein were determined via western blot to analyze the effect of siRNAs on tumor cell apoptosis.

**Results:**

The anti-tumor effect appeared in all groups with siRNA-mediated inhibition. The tumor growth suppression was stronger in the group with double inhibition. The weight and volume of the tumors were significantly lower and the survival rate improved in the three siRNA groups. IFN-γ levels increased but IL-10 levels decreased in the blood of the siRNA group mice compared with the results for the control group. In the tumor and spleen tissue, the IFN-γ levels significantly increased, but in the liver tissue they significantly decreased in the three siRNA groups. The results of quantitative RT-PCR showed that the mRNAs for *PD-1* and *CTLA-4* were downregulated in spleen tissue in the three siRNA groups, while the *PD-L1* mRNA and protein levels increased significantly in the tumor, but decreased in the liver. *Survivin* and *IL-6* mRNA levels decreased in the tumor. Western blot results showed that ratio of Bax and Bcl-2 had significantly increased. These results indicated that downregulating *PD-1* and *CTLA-4* could increase the body’s immune response and promote apoptosis of tumor cells.

**Conclusion:**

Co-inhibiting the expressions of *PD-1* and *CTLA-4* can effectively suppress the growth of H22 hepatoma and promote the apoptosis of tumor cells in mice. Blocking PD-1 and CTLA-4 can improve the vitality of T cells, and improve the immune environment and response.

## Introduction

Cancer cells can often evade the immune system. The main factor enabling this escape ability is the presence of negative regulatory receptors on the T-cell surface [1]. Programmed cell death protein-1 (PD-1) and cytotoxic T lymphocyte antigen 4 (CTLA-4) are the most important of these negative regulatory receptors [2].

PD-1 is a member of the CD28 family. It is a type I transmembrane protein expressed on the surface of activated T-cell membranes [3]. The main ligands of PD-1 are PD-L1 (programmed cell death-ligand1) and PD-L2 (programmed cell death-ligand 2), which can interact with PD-1 to produce inhibitory signals, inhibit the activation and proliferation of T cells, and reduce the immune response to tumor cells [4].

CTLA-4 is a transmembrane protein encoded by the *CTLA-4* gene, expressing in activated CD4^+^ (auxiliary) and CD8^+^ (cytotoxic) T cells. By binding with its ligand B7 molecules, CTLA-4 produces inhibitory signals and inhibits the activation of T cells [5–7] to protect the tumor cell from attack.

Therefore, blocking the ligand binding site or reducing the expression of PD-1 and CTLA-4 should stimulate the proliferation of immune cells and induces or enhances the anti-tumor immune response. A large number of experiments have proved this assumption. Blocking the PD-1/PD-L1 signaling pathway inhibited the growth of a variety of malignant tumors [8], and downregulation of CTLA-4 also showed great potential for tumor suppression.

Preclinical and clinical studies also show that the immune checkpoint therapy provides a survival benefit for some significant number of patients with liver cancer, and a combination of anti-PD-1/PD-L1 and anti-CTLA-4 antibodies is an effective treatment strategy forhepatocellular carcinoma (HCC) [9, 10].

The usual method of blocking these signaling pathways is with an antibody or small chemical molecule. In this study, double-stranded interference RNA (siRNA) is applied to inhibit the expression of PD-1 and CTLA-4 to study the feasibility of siRNA as a therapeutic for liver cancer.

## Experimental materials and methods

### Animals and tumor cell lines

All the mice in study were male ICR (Institute of Cancer Research) mice, purchased from Beijing Vital Lihua with Certificate of Quality No. SCXK 2016–0011. They weighed 22–27 g at the time of purchase. The animals were housed individually in cages in a temperature-controlled room with a 12-h light/dark cycle. After one week of acclimation with free access to regular rodent chow and water, the mice were used for further experiments.

The H22 hepatocarcinoma cells were donated by Weifang Medical College. All the animal experimental procedures in this study were conducted in accordance with protocols approved by the Institutional Ethical Committee of Qingdao Medical University.

### Modeling tumor-bearing mice

H22 hepatocarcinoma cells were injected into the peritoneal cavities of 48 mice and passaged three times in succession. Ascite buildup was visible 7–9 days later. The viscous ascite was extracted and the milk-white ascite was selected for the cell count. It was diluted to give a cell count of 1 × 10^7^/ml. Each mouse was inoculated with 0.2 ml of the cell suspension into the right forelimb armpit [11].

### Introduction of siRNA

After the tumor had grown for 6 days, the mice were randomly divided into four equal groups (*n* = 12): control, siPD-1, siCTLA-4 and siPD-1+ siCTLA-4 groups. Each group was given a different transfection reagent (Entranster-in-vivo, EngreenBiosystem Co., Ltd.) and different siRNA: negative control siRNA, siPD-1, siCTLA-4, or siPD-1 + siCTLA-4 (synthesized by Shanghai GenePharma Co., Ltd.). The transfection reagent (about 30 μl) and 19.8 μg siRNA were injected into each tumor once every 3 days for one month. Twenty-four hours after the last administration of the tested drug on day 30 of the experiment, blood samples were collected from the mice’s eyes. The serum was harvested by centrifugation. Then, all the mice were euthanized.

The sequence of siPD-1: sense:5′-CCUGGAGACCUCAACAAGAdTdT-3′, antisense: 5′-UCUUGUUGAGGUCUCCAGGdTdT-3′. The sequence of siCTLA-4: sense: 5′-GAUCCUUGUCGCAGUUAGCdTdT-3′, antisense: 5′-GCUAACUGCGACAAGGAUCdTdT-3′.The sequence of negative control siRNA: sense: 5’-UUCUCCGAACGUGUCACGUdTdT-3′, antisense: 5′-ACGUGACACGUUCGGAGAAdTdT-3′.

### Observing the physiological condition of the H22 mice

The physiological condition of mice was assessed once every 4 days during the experiment. This included measuring the volume of the solid tumor,the mass of food intake, and the survival time. The calculation of the solid tumor volume was:

$$ \mathrm{V}=1/2\times {\left(\mathrm{a}\times \mathrm{b}\right)}^2 $$

where a and b represent the largest and the smallest diameters, respectively. The calculation of tumor growth inhibition rate was:

$$ \left(\mathrm{Control}\ \mathrm{tumor}\ \mathrm{weight}-\mathrm{Experimental}\ \mathrm{group}\ \mathrm{tumor}\ \mathrm{weight}\right)/\mathrm{Control}\ \mathrm{group}\ \mathrm{tumor}\times 100\% $$

The test continued for 30 days. Animals that lived more than 30 days were defaulted as 30 days. The survival rate was calculated as a percentage:

$$ \mathrm{Survival}\ \mathrm{rate}=\left\{\left(12\hbox{-} \mathrm{number}\ \mathrm{of}\ \mathrm{mice}\ \mathrm{that}\ \mathrm{died}\ \mathrm{in}\ \mathrm{each}\ \mathrm{group}\right)/12\right\}\times 100\% $$

### Determining the levels of IFN-γ, IL-10 and PD-L1

After siRNAs were injected for one month, blood was taken from the eyeball of the mice and kept at room temperature for 1 h to precipitate the blood cells. The serum (supernatant) was obtained by centrifugation at 5000 rpm for 10 min.

The interferon gamma (IFN-γ) and interleukin 10 (IL-10) levels in the serum were determined using an ELISA kit according to the manufacturer’s instructions (cat. no. ELM-IFNg and ELM-IL10, RayBiotech).

The PD-L1 protein level in the tumor and liver tissue was also assayed via ELISA. Samples of 100 mg tumor or liver tissue were taken from each mouse, washed bloody with phosphate buffer saline (PBS), and then placed in 1 ml PBS for homogenization. The homogenate was stored at − 20 °C overnight, then centrifuged at 1000 g for 10 min. The precipitate was discarded and the supernatant was retained. The PD-L1 protein in the supernatant was assayed with an ELISA kit according to the manufacturer’s instructions (cat. no. CSB-EL004911MO, Wuhan Huamei Biotechnology Co., Ltd.).

### Determining the mRNA levels of *IFN-γ*, *PD-1*, *CTLA-4*, *Survivin* and, *IL-6*

Quantitative RT-PCR was used to determine the mRNA expression levels of *IFN-*γ, *IL-6*, *PD-L1* and *Survivin* in the tumor, *IFN-γ*, *PD-L1* in the liver and *IFN-*γ, *PD-1* and *CTLA-4* in the spleen. Tissue samples were taken. The tissue was partly excisedand total RNA was extracted with Trizol reagent (Takara Company). cDNA was obtainedwith a reverse transcription kit (TransGen Biotech), amplified via quantitative PCR (TransGen Biotech). The reaction conditions were 94 °C for 30s, 94 °C for 5 s and 60 °C for 30s, for forty cycles. Three parallel reactions were performed for each sample. The 2^-△△Ct^ method was used to calculate the expression of each mRNA in each mouse. The primer sequences are shown in Table 1.

**Table 1:** Primer sequences for PCR

Gene name	Primer sequence
*β-actin*	Sense: 5′-ATGGGTCAGAAGGACTCCTATG-3′ Antisense: 5′-ATCTCCTGCTCGAAGTCTAGAG-3′
*PD-1*	Sense: 5′-GCCTGGCTCACAGTGTCAG-3′ Antisense: 5′-TCCAGGGCTCTCCTCGATT − 3′
*CTLA-4*	Sense: GTCTTCTCTGAAGCCATACAG −3′ Antisense: 5′-GACCTCATCAGTGTTGTGTGA-3′
*PD-L1*	Sense: 5′-GGAATTGTCTCAGAATGGTC-3′ Antisense: 5′-GTAGTTGCTTCTAGGAAGGAG-3′
*IFN-γ*	Sense: 5′-GCTTTGCAGCTCTTCCTCAT-3′ Antisense: 5′-GTCACCATCCTTTTGCCAGT-3′
*IL-6*	Sense: 5′-CTTAATTACACATGTTCTCTGGGAAA-3′ Antisense: 5′-CAAGTGCATCATCGTTGTTCATAC-3′

### Determining the expressions of Bax and Bcl-2

Western blot was used to determine the expression levels of Bax and Bcl-2. Samples of tumor tissue (50 mg) were taken from each mouse and put in 400 μl of RIPA buffer with 1% PMSF. The protein was extracted using aconventional method. The concentration of protein was determined via the BCA method and adjusted to the same concentration for every sample.

30 μg (about 6 μl) of protein was separated using 15% SDS-PAGE, then transferred onto PVDF membrane under 300 mA of current for 2 h. The membrane was blocked with 5% skimmed milk at room temperature for 2 h, thenincubated with the primary antibodies (anti-Baxdiluted 1:200 andanti-Bcl-2diluted 1:200, Santa Cruz Biotechnology, Inc.; and anti-β-actindiluted 1:700, Zhongshan Bio-Tech Co., Ltd.) at 4 °C overnight. The membrane was taken out, washed with TBST 3 times, and incubated withthe secondary antibody (anti-mouse antibody, diluted 1:7000, MultiSciences) at room temperature for 1 h, followed by 3 washes with TBST, each for 10 min.

Through the development of ECL hypersensitive luminescent liquid (chemiluminescent HRP Substrate), the gray value of the gel image was analyzed using the appropriate software. The expressions of Bax and Bcl-2 were determined based on the ratio of the Bax and Bcl-2 protein gray values.

### Statistical analysis

The results are expressed as means ±SD. The differences were determined using the ANOVA test, except for the survival rate, which was determined using the Logrank-test.A value less than 0.05 (*p* < 0.05) was considered statistically significant. The statistical analyses were performed using the GraphPad Prism 6.0 statistical software package.

## Results

### Physiological observations

In the first 5 days after inoculation with H22 cells, the mice maintained a good living condition. Their hair remained bright in color and there was no significant difference between the groups. After 5 days, small nodules appeared in all the groups of mice. As the experiment progressed, the tumor in the single siRNA groups grew more slowly than in the control group. The slowest growth was seen in the *PD-1 + CTLA-4* siRNA group. The mice in the control group displayed a gradual dimming of hair color and loss of appetite. They also exhibited abnormal activity.

### The inhibitory effect of siRNA on the tumor

**Fig. 1:**
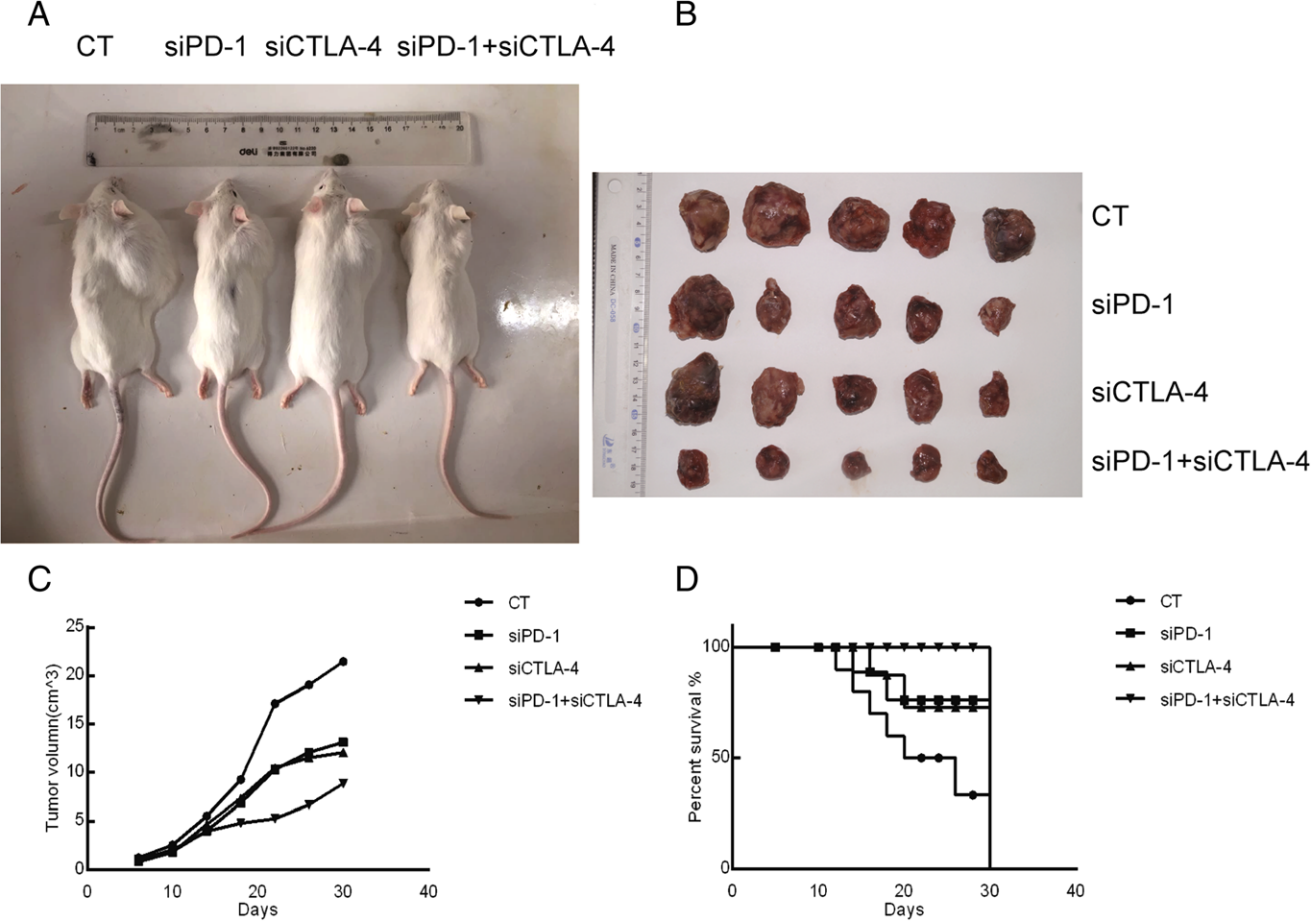
The antitumor effect of siPD-1 and siCTLA-4 in the H22 allograft. CT = control, siPD-1 = group injected with siRNA for PD-1, siCTLA-4 = group injected with siRNA for CTLA-4, siPD-1 + siCTLA-4 = group injected with siRNA for PD-1 + CTLA-4. **a** –Photo of four representative tumor-bearing mice (one per group). The picture was taken 30 days after inoculation with H22 cells. **b** – Photo of 5 solid tumors from each group. Tumors were removed from mice 30 days after inoculation with H22 cells. **c** – Change curve of tumor volume. Tumor diameter was measured with a caliper once every 4 days for 30 days and volume was calculated as described in section observing the physiological condition of the H22 mice 2.4. **d** – Survival rate of mice. These results indicate that siRNAs markedly inhibited the growth of tumor

The volumes and weights of the tumor in the siRNA groups were significantly below those of the control group, with the most pronounced effect in the double siRNA group (*p* < 0.05). The results are shown in Table 2 and Fig. 1.

**Table 2:** The tumor inhibition rate for each siRNA (mean ± SD, *n* = 12)

Group	N	Tumor weight (g)	Inhibition rate (%)
Control	12	6.9075 ± 2.71	–
siPD-1	12	1.7067 ± 0.65*	75.29%
siCTLA-4	12	1.7144 ± 0.39*	75.18%
siPD-1 + siCTLA-4	12	0.7440 ± 0.15*	89.23%

*p < 0.05 vs.the control group

### The effect of siRNA on IFN-γ, IL-10 and PD-L1 levels in the blood and tissue

**Fig. 2:**
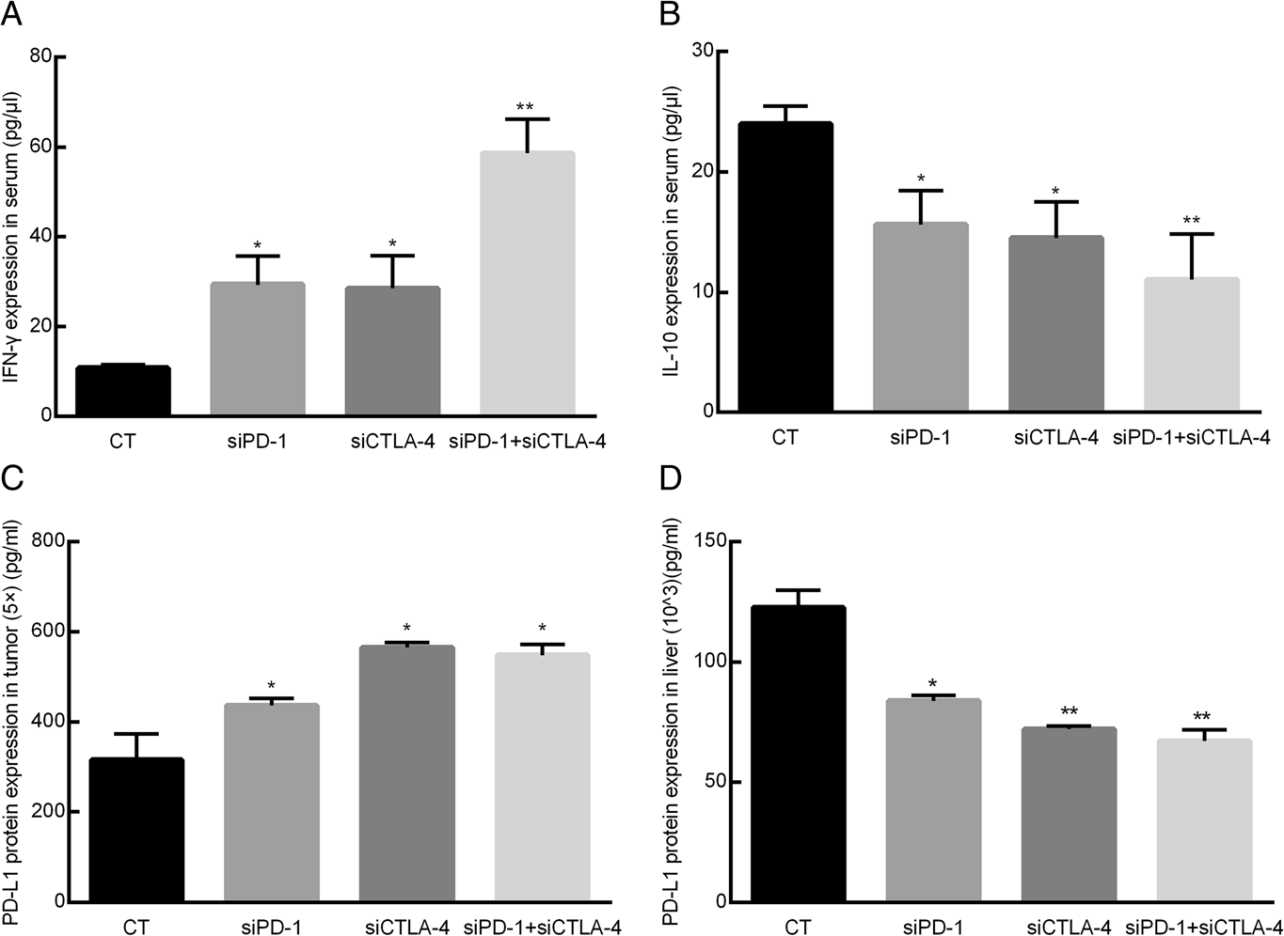
IFN-γ, IL-10 and PD-L1 levels in the blood and tissue. These results are based on ELISA tests. CT = control, siPD-1 = group injected with siRNA for PD-1, siCTLA-4 = group injected with siRNA for CTLA-4, siPD-1 + siCTLA-4 = group injected with siRNA for PD-1 + CTLA-4. **a** – IFN-γlevel in mouse serum. **b** – IL-10 level in mouse serum. **c** – PD-L1 level in tumor tissue. **d** – PD-L1 content in liver tissue. The expressions of IFN-γ, IL-10 and PD-L1 in the blood and tissue of the siRNA groups were significantly different from those in the control group (**p < 0.01, *p < 0.05)

ELISA was employed to analyze the levels of IFN-γ, IL-10 and PD-L1. The results show that the IFN-γ level in the blood of the siRNA group mice had increased, while the level of the cytokine IL-10 had significantly decreased (*p* < 0.05) compared with the control group. The PD-L1 level had significantly increased in the tumor, but significantly decreased in the liver compared with the control group (*p* < 0.05, Fig. 2).

### The effect of siRNA onthe mRNA of *IFN-γ, PD-L1, PD-1, CTLA-4*, *IL-6* and *Survivin*in the tissue

The effects of siRNAs on the mRNA of *IFN-γ, PD-1, PD-L1, CTLA-4* and *Survivin* in the tumor, liver and spleen were determined using quantitative RT-PCR. The results showed that the mRNA levels of *IFN-γ* and *PD-L1* in the tumor of the siRNA groups had significantly increased compared with the control group (*p* < 0.05).*Survivin* and *IL-6* expression had decreased (*p* < 0.05). Compared with the control group, the expressions of *PD-L1* and *IFN-γ* in the liver had decreased (*p* < 0.05). The mRNA levels of *PD-1* and *CTLA-4* had decreased, but *IFN-γ* had increased in the spleen (*p* < 0.05) compared with the control group.

**Fig. 3:**
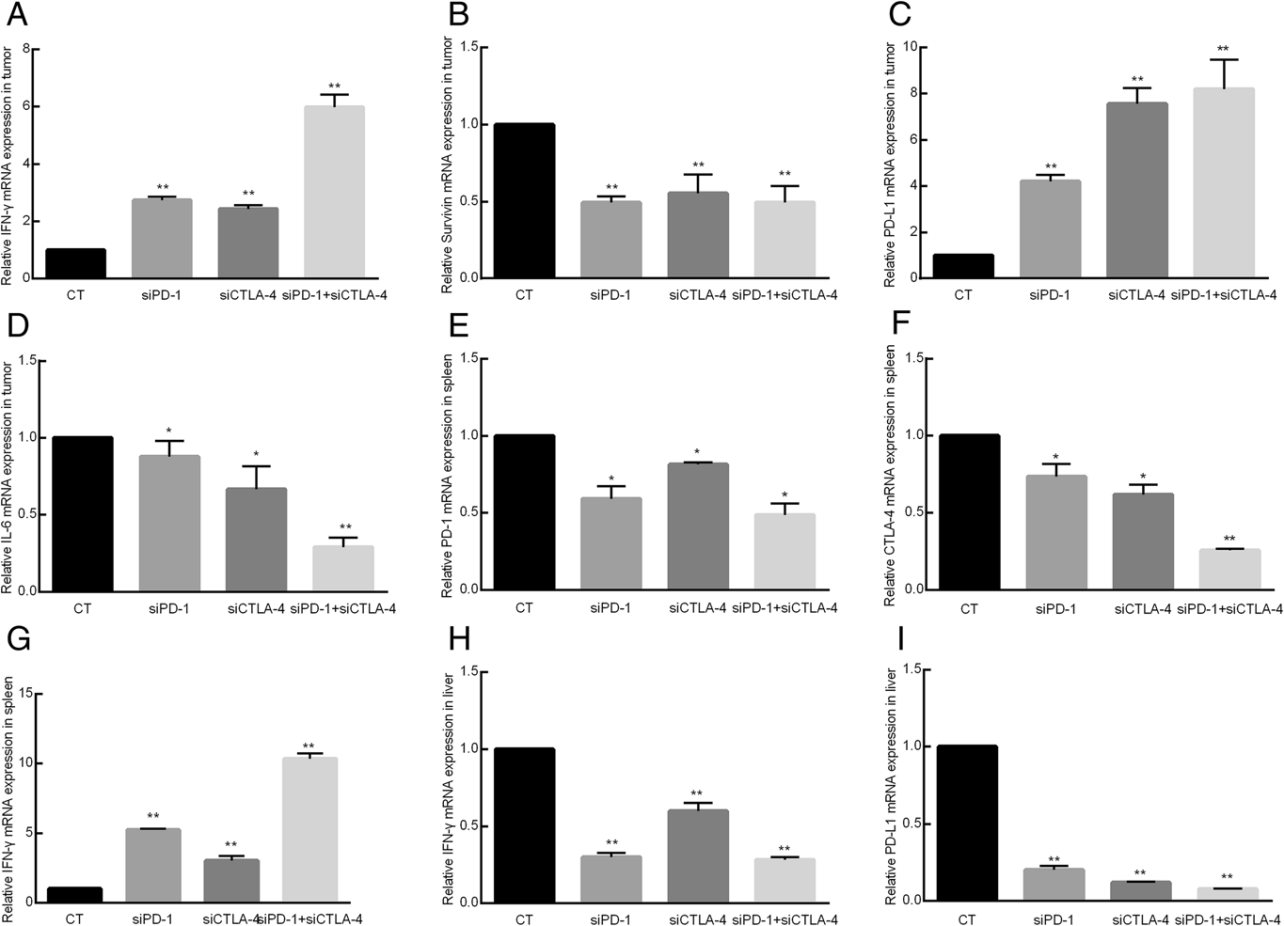
PCR results for *PD-1*, *PD-L1*, *CTLA-4*, *IFN-γ*, *IL-6* and *Survivin* mRNA in various tissues. CT = control, siPD-1 = group injected with siRNA for PD-1, siCTLA-4 = group injected with siRNA for CTLA-4, siPD-1 + siCTLA-4 = group injected with siRNA for PD-1 + CTLA-4. **a** – Level of *IFN-γ* mRNA in the tumors. **b** – Level of *Survivin* mRNA in the tumors. **c** – Level of *PD-L1* mRNA in the tumors. **d** – Level of *IL-6* mRNA in the tumors. **e** – Level of *PD-1* mRNA in the spleen. **f** – Level of *CTLA-4* mRNA in the spleen. **g** – Level of *IFN-γ* mRNA in the spleen. **h** – Level of *IFN-γ* mRNA in the liver. **i** – Level of *PD-L1* mRNA in the liver. The mRNA expressions of some genes in the siRNA groups were significantly different than those in the control group (*p < 0.05, **p < 0.01)

These results indicate that *PD-1* siRNA and *CTLA-4* siRNA could enhance the immune response of mice and inhibit the expressions of some oncogenes. Interestingly, the expression of *PD-L1* increased in the tumor. This phenomenon may be attributed to a compensation effect in the organism. When PD-1 was downregulated, the tumor cells attempted to utilize the PD-1/PD-L1 pathway to escape the immune cell attack and express more PD-L1, so the mRNA level of PD-L1 increased (Fig. 3).

### The effect of siRNA on Bax and Bcl-2 in the tumor

**Fig. 4:**
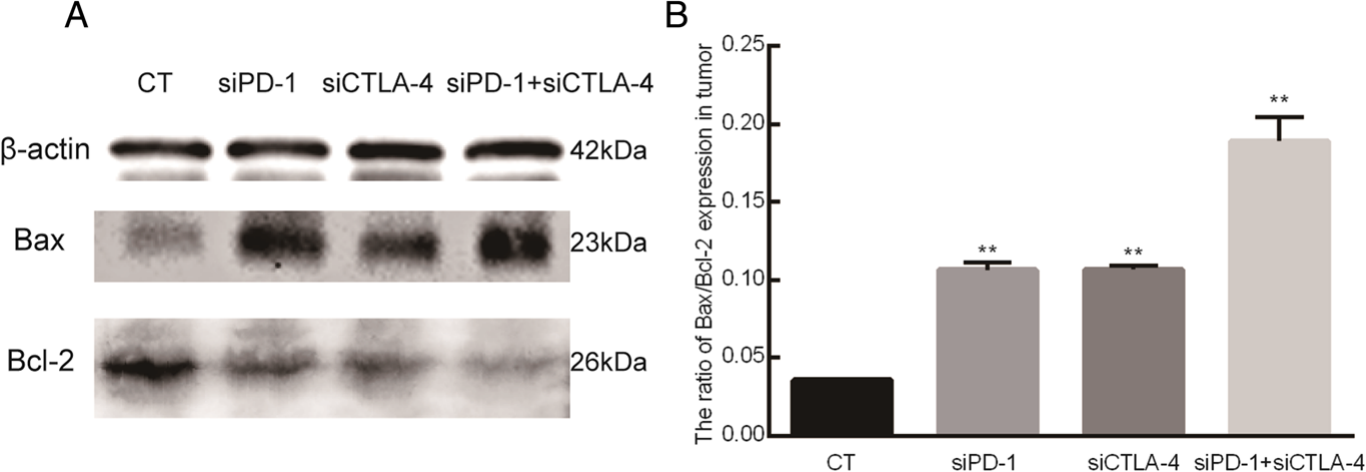
Bax and Bcl-2 assays. The levels of Bax and Bcl-2 were determined using western blot. **a** – Photo of the western blot gels for the four tumor samples. CT = control, siPD-1 = group injected with siRNA for PD-1, siCTLA-4 = group injected with siRNA for CTLA-4, siPD-1 + siCTLA-4 = group injected with siRNA for PD-1 + CTLA-4. **b** – Quantitative analysis of Bax and Bcl-2 expressions. Compared with the control group, the Bax-to-Bcl-2 ratio significantly increased in all siRNA groups (**p < 0.01, *p < 0.05)

The tumor Bax and Bcl-2 levels were determined via western blotting. The ratio of Bax to Bcl-2 increased in siPD-1, siCTLA-4 and siPD-1 + siCTLA-4 groups (*p* < 0.05) compared to the levels for the control group (Fig. 4).

## Discussion

PD-1 is mainly expressed in activated T cells, where its function is to inhibit their excessive activation. This is a normal self-stabilizing mechanism of the immune system [12]. Its ligand, PD-L1, is mainly expressed on the surface of tumor cells [13]. The tumor microenvironment can induce infiltrating T cells to express PD-1. When PD-1 and PD-L1 interact, the PD-1/PD-L1 signaling pathway maintains the activation. In this case, the activation of T lymphocytes is inhibited, and tumor cells escape immune surveillance [14–16]. siRNA can block the PD-1 pathway, partially restoring the function of T cells, so that they can continue to kill tumor cells [17].

Both CD28 and CTLA-4 are expressed on the surface of T cells. They competitively bind with CD80 (B7–2) and CD86 (B7–1) [18], but their functions are distinct. The main function of CD28 is to participate in the activation of T cells [19]. CTLA-4 is an immunosuppressive receptor, but it has a stronger affinity with the two ligands than CD28 [20].

The interaction of CTLA-4 and its ligands generates an inhibitory signal and decreases or represses the viability of T cells [21].Blocking the interaction of CTLA-4 and ligandspromotes CD28 to bind the two ligands, activates T cells, improves the body’s immune response, and kills tumor cells. An existing study shows that inhibiting the binding of CTLA-4 and its ligands also can downregulate Bcl-2 and promote the apoptosis of tumor cells.

IFN-γ is produced by T cells. It can directly inhibit the proliferation of tumor cells [22], increase the expression of surface MHC antigen and tumor necrosis factor (TNF), and play an important role in anti-tumor angiogenesis. IFN-γ can also regulate the expression of Fas/FasL in tumor cells and enhance the sensitivity of tumor cells to the Fas-mediated apoptosis pathway, reducing the ability of tumor cells to evade attack by the immune system. Therefore, enhancing T-cell activity increases the release of IFN-γ and produces astronger inhibitory effect on the tumor [23].

IL-10 is a cytokine that regulates immune function, acting on a variety of immune cell subsets and playing a role in immune suppression in a variety of ways that can cause or prevent tumor immune escape. Recent studies reported that IL-10 also stimulates the immune response, suggesting that it is a multifunctional cytokine with positive and negative regulatory effects [24].

IL-6 is a multifunctional inflammatory cytokine with an important role in inflammation and tumorigenesis. It is also an important tumor-promoting cytokine that enhances proliferation and anti-apoptotic effects in tumor cells [25]. Furthermore, IL-6 levels in cancer tissues and serum are elevated in HCC patients. They correlate with tumor metastasis and reduced patient survival rate [26]. The expression of IL-6 mRNA decreased in our experiment, leading to an inhibition of proliferation and increase in apoptosis of tumor cells.

Our study mainly applied RNAi technology with the aim of blocking the two signaling pathways. PD-1/PD-L1and CTLA-4/CD80, /CD86 were used to enhance the activity of T cells to inhibit the growth of tumor cells. Mice were inoculated with H22 tumor cells to yield the tumor model, and then siRNAs were injected into the tumor.

Our results indicate that the siRNAs blocking the two signaling pathways could significantly improve the immune response in the mice. They show that tumor growth was significantly inhibited and that the expression of PD-1 and CTLA-4 in the tumor tissue significantly decreased after the introduction of siPD-1 and siCTLA-4. The expressions of IFN-γ obviously increased in the blood, tumor and spleen in the siRNA groups, but decreased in the liver. The IL-10 level in the blood decreased in the siRNA groups. The reduction in the *IFN-γ* mRNA level in the livers of the siRNA group mice could be a protective effect of siRNA on the liver tissue, reducing the inflammation reaction.

The expressions of PD-L1 in the livers of siRNA group mice were significantly lower than those for the control group mice. The expressions of PD-L1 were mainly on the surface of the tumor cells, rather than in the normal tissue. In this study, there was a high expression level in control group, but very low in the siRNA groups. The results indicated that H22 hepatoma cells had metastasized to the liver in the control group, but not in the siRNA groups. siRNAs blocking the PD-1/PD-L1 or CTLA-4/CD80, /CD86 pathways could reduce the metastasis of tumor cells.

Interestingly, when the PD-1 signaling pathway was blocked, the expression of PD-L1 increased in the tumor. This phenomenon may be due to the tumor cells attempting to employ PD-1/PD-L1 signaling pathway to escape the attack by the immune cells and enhance the expressions of PD-L1. This would increase the mRNA and protein levels of PD-L1. This is a compensation effect.

*Survivin* is an inhibitor of apoptosis. It only expresses in tumor and embryonic tissues [27]. *Survivin* is closely related to the differentiation, proliferation, invasion and metastasis of tumor cells [28]. The results of this study indicate that the expression of *survivin* in the siRNA groups was significantly lower than that in the control group. The reduction in the *surviving* level was consistent with the promotion of tumor cell apoptosis.

Bax and Bcl-2 are a pair of important apoptosis-related proteins in the body. Any increase in the level of Bax or decrease in the level of Bcl-2 can promote tumor cell apoptosis, so Bax/Bcl-2 clearly have a close relationship to the genesis and development of tumors [29]. Our results show that the level of Bcl-2 was lower and that of Bax was higher when *PD-1* or *CTLA-4* were downregulated. This result is consistent with previous reports and it proves again that blocking the PD-1/PD-L1 or CTLA-4/CD80, /CD86 pathways could promote the apoptosis of tumor cells [30].

Overall, downregulation of PD-1 or CTLA-4 had a significant inhibitory effect on the growth of the tumor. Double downregulation of PD-1 and CTLA-4 had a more obvious effect than single gene downregulation.

Currently, most targeted immunotherapy drugs are antibodies or small molecule drugs [31]. Small molecule drugs are generally chemical substances that are difficult to metabolize into nontoxic water and CO_2_ and can have toxic side effects on the body. Antibody-based immunotherapies have some disadvantages, such as their high cost, limited half-life, and immunogenicity. Our aim is to find an alternative method to effectively inhibit the proliferation of tumor cells but with fewer side effects.

siRNA synthesis is easy, low cost, and will not lead to an immune response. On the other hand, siRNA is a biological molecule, easy to metabolize, and has no side effect on the body. Our results indicate that siRNA can be applied to inhibit tumor growth and could be developed into an RNAi-based immunotherapy or even a precision medicine for tumors.

## Conclusions

In the study, we knocked down the expressions of PD-1 and CTLA-4 using siRNA. Our result show that the growth of tumor cells was significantly inhibited in siRNA groups. When the two signaling pathways of PD-1/PD-L1 and CTLA-4/CD80, /CD86were blocked, the vitality of T cells was enhanced, and the antitumor immune response of the body increased. This promoted the apoptosis of H22 hepatoma cells and reduced the metastasis of tumor cells. Simultaneous knockdown of PD-1 and CTLA-4 to inhibit proliferation of H22 hepatoma cell was more effective than single knockdown of PD-1 or CTLA-4 and did not appear to be toxic. Co-inhibiting oncogene expression is an attractive potential treatment for cancer.

## Abbreviations

CD4+/CD8 +: Auxiliary T cells/cytotoxic T cells
CTLA-4: Cytotoxic T lymphocyte antigen 4
ELISA: Enzyme-linked immunosorbent assay
IFN-γ: Interferon gamma
IL-10: Interleukin 10
IL-6: Interleukin-6
PD-1: Programmed cell death protein-1
PMSF: Protease inhibitor
RIPA: Rapid cell and tissue lysate
siRNA: Double-stranded interference RNA
siPD-1: Interference RNA PD-1
siCTLA-4: Interference RNA CTLA-4
SDS-PAGE: sodium dodecyl sulfate Polyacrylamide gel electrophoresis
